# Molecular Identification of *Leishmania* Species in a Re-Emerged Focus of Cutaneous Leishmaniasis in Varamin District, Iran

**Published:** 2017-03-14

**Authors:** Mahmoodreza Behravan, Vahideh Moin-Vaziri, Ali Haghighi, Nourina Rahbarian, Niloofar Taghipour, Alireza Abadi, Homa Hajjaran

**Affiliations:** 1 Department of Medical Parasitology and Mycology, School of Medicine, Shahid Beheshti University of Medical Sciences, Tehran, Iran; 2 Cellular and Molecular Biology Research Center, Shahid Beheshti University of Medical Sciences, Tehran, Iran; 3 Department of Social Medicine and Health, School of Public Health, Shahid Beheshti University of Medical Sciences, Tehran, Iran; 4 Department of Medical Parasitology and Mycology, School of Public Health, Tehran University of Medical Sciences, Tehran, Iran

**Keywords:** Cutaneous leishmaniasis, PCR-RFLP, *Leishmania tropica*, *Leishmania major*, Iran

## Abstract

**Background:**

Cutaneous leishmaniasis (CL) is one of the most important neglected tropical diseases and a major public health challenge in Iran caused by *Leishmania* spp and transmitted by phlebotomine sand flies. The number of CL cases has shown an increasing pattern all over the country, including the district of Varamin, southeast of Tehran, Iran. This study aimed to identify the *Leishmania* spp isolated from CL patients using molecular methods in Varamin during 2012–2013.

**Methods:**

Exudate materials collected from the swollen edge of the skin lesions of 44 parasitological positive CL patients by disposable lancet. They were referred to Varamin Health Center by physician. The samples were subjected to molecular method for *Leishmania* species identification.

**Results:**

The digestion pattern of restriction enzyme revealed that 37 (84.1%) CL patients were infected with *L. major* and 7 (15.9%) were infected with *L. tropica*. They were mostly male than female. More than half of the patients (58%) had multiple lesions, and they were mostly observed on extremities, 34.1% on legs and 29.5% on hands. Lesions were mostly of wet ulcerative type.

**Conclusion:**

Dominancy of *L. major* provides more evidence that Varamin District probably could be considered as Zoonotic Cutaneous Leishmaniasis (ZCL) areas. More investigation on other epidemiological aspects of disease is needed.

## Introduction

Leishmaniasis is one of the most devastating neglected tropical diseases with a complicated ecology (Alvar et al. 2012). Disease is a sand fly borne infection caused by the blood parasite *Leishmania*, more than 20 species of parasites are transmitted via the infective bites of different species of sand flies (Subfamily Phlebotominae) (Alvar et al. 2012). Leishmaniasis has been clinically categorized into three main forms: visceral (VL), cutaneous (CL), and mucocutaneous (MCL) (Reithinger et al. 2007). Endemic transmission of the disease has been reported from different tropical and subtropical countries with 350 million people at risk, the overall prevalence is about 12 million worldwide with yearly estimated incidence of 0.2–0.4 million cases of VL and 0.7–1.2 million cases of CL (Alvar et al. 2012).

Iran is a main endemic region of CL in the Middle East and North Africa (McDowell et al. 2011). Cutaneous leishmaniasis is endemic in two forms, Anthroponotic Cutaneous Leishmaniasis (ACL) and Zoonotic Cutaneous Leishmaniasis (ZCL). Anthroponotic form is still of great importance in many parts of the country, including some large and medium sized cities such as Tehran, Mashhad, Neishabur and Sabzevar in the north-east, Shiraz in the south, Kerman and Bam in the southeast, Yazd, Kashan and parts of the city of Esfahan in the central region (Yaghoobi-Ershadi et al. 2010, Yaghoobi-Ershadi 2012). The parasite is *L. tropica* and the vector is supposed to be *Phlebotomus* (*Paraphlebotomous*) *sergenti* Parrot 1917. The main reservoir host is human but dogs have a role as animal reservoir host (Yaghoobi-Ershadi 2012). Zoonotic form is endemic in many rural areas of 17 out of 31 provinces of Iran (Yaghoobi-Ershadi 2012). Rodents are considered as the main reservoirs of disease, *Rhombomys opimus* is the main animal reservoir in foci located in the north-east and central part of the country, *Meriones libycus* in some parts of central and south of the country, *Tatera indica* in the southeast and *M. hurrianae* in southeastern part of Baluchistan, neighboring Pakistan (Akhavan et al. 2010, Azizi et al. 2012, Yaghoobi-Ershadi 2012, Hajjaran et al. 2014). *Phlebotomus papatasi* Scopoli 1786, the most prevalent species among *Phlebotomus* genus, is the only known vector (Yaghoobi-Ershadi 2012).

From 1983 to 2012, cases of all types of leishmaniasis in Iran reached to 569164, which 99.5% of that was CL. In 2012, totally 20947 cases were recorded all through the country and highest incidence rate was observed in Ilam, Fars and Khorassan Razavi provinces in that year (Shirzadi et al. 2015).

Official reports showed an increasing trend in number of CL cases in Varamin District, from 18 cases in 2010 to 32 cases in 2011 (Communicable disease report, unpublished data). Varamin is located 35km, southeast of Tehran, Iran. A purely parasitological study was done in Varamin by the same authors (Behravan et al. 2015), but parasite identification at species level was not done. *Leishmania* species are not morphologically distinguishable.

Identification of *Leishmania* parasites is essential for precise prognosis of the disease as well as making proper decision regarding control and probably treatment (Hajjaran et al. 2014). Therefore, this study was conducted to characterize the causative agents of disease by molecular tools. In recent years, different molecular methods with different genetic markers have been developed for parasite identification (Schonian et al. 2003, Parvizi et al. 2008, Parvizi and Ready 2008, Azizi et al. 2012). Internal Transcribed Spacer-1 (ITS1) of the small subunit ribosomal DNA was known as a reliable marker for detection of *Leishmania* parasite. Different digestion patterns which were produced by HaeIII (BsuRI) enzyme can clearly differentiate the parasite (Hajjaran et al. 2011, Al-Nahhas and Kaldas 2013, Hajjaran et al. 2013). This method also was used in current study to identify *Leishmania* species in CL suspected patients in Varamin district.

## Materials and Methods

### Study Area

**Fig. 1.**
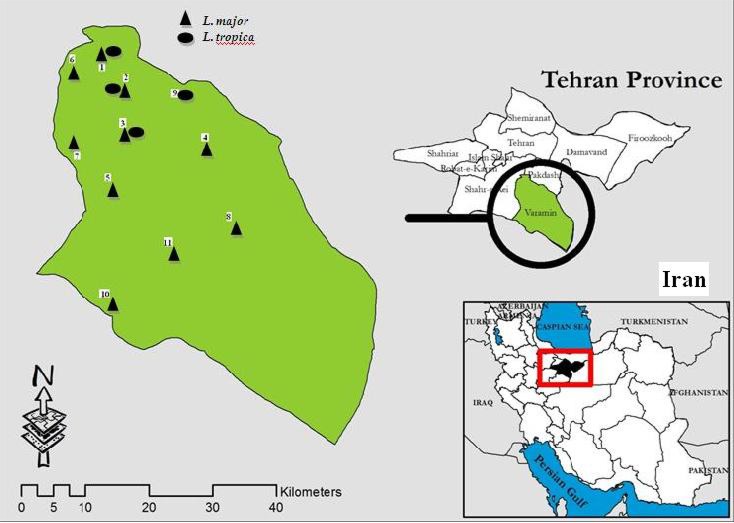
Geographical location of Varamin, (inset is map of Iran), showing the study area and distribution of *Leishmania major* and *Leishmania tropica* isolated from patients during 2012–2013. Numbers on the map refer to the villages of: 1: Bagher-Abad, 2: Kheir-Abad, 3: Varamin, 4: Deh-Sharifa, 5: Ahmad-Abad, 6: Vali-Abad, 7: Mohammad-Abad, 8: Omr-Abad, 9: Reyhan-Abad, 10: Charm-Shahr, 11: Javad-Abad

The present study was conducted in Varamin (35°19′27″N, 51°38′45″E), located 35 km southeast of Tehran, Iran (Fig. 1).

### Sampling and DNA extraction

This study was done on CL suspected patients who were referred to Varamin Health Center laboratory during 2012–2013 from 11 different areas mentioned in Figure and Table 1. Patient’s information (age, sex, living area, ulcer duration and number and site of lesions) was recorded by a special questionnaire.

**Table 1.** Number of cases and distribution of cutaneous leishmaniasis agents based on study areas, Varamin District, Iran, 2012–2013

**Study area**	**Cases No. (%)**	**PCR-RFLP results No.**	

		*L. major*	*L. tropica*
**Bagher-Abad**	12(27.3)	10	2
**Kheir-Abad**	10(22.7)	8	2
**Varamin**	8(18.2)	6	2
**Deh-Sharifa**	3(6.8)	3	0
**Ahmad-Abad**	2(4.5)	2	0
**Vali-Abad**	2(4.5)	2	0
**Mohammad-Abad**	2(4.5)	2	0
**Omr-Abad**	2(4.5)	2	0
**Reyhan-Abad**	1(2.3)	0	1
**Charm-Shahr**	1(2.3)	1	0
**Javad-Abad**	1(2.3)	1	0
**Total**	44 (100)	37	7

Ethic clearance was obtained from Research Ethical Committee of Shahid Beheshti University of Medical Sciences (Approval Number: sbmu.rec.1392.253).

Smears were prepared from exudate materials of swollen edge of lesions of patients collected by sterile lancet, then fixed in methanol, stained by Giemsa and examined under a light microscope for the presence of amastigotes. Grading of *Leishmania* parasites was obtained by average parasite density as follows: 4+ (1–10 parasites/fields), +3 (1–10 parasites/10 fields), 2+ (1–10 parasites/100 fields), 1+ (1–10 parasites/1000 field) according to WHO protocols (WHO 1991). Exudate materials from ulcer of CL suspected patients also were used for molecular identification. DNA was extracted by using the Bioneer DNA Extraction Kit (Bioneer, Republic of Korea) in accordance with the manufacturer’s instructions. DNA extracts were stored at −20 °C until being used.

### ITS1 amplification and enzymatic digestion

ITS1 was amplified by using specific primers, LITSR (forward: 5′-CTGGATCATTTT CCGATG-3′) and L5.8S: (reverse: 5′-TGA TACCACTTATCGCACTT-3′). The amplification was carried out by using the PCR-Ready premix (Roche, Germany) in 25μl total reactions comprising 10 μl premix, 2μl forward and reverse primers (10 pmol), 1μl DNA template and 13μl double distilled water. Iranian reference strains of *L. major* (Accession Number: JN860745) and *L. tropica* (Accession. Noumber: EF653267) were used as positive standard controls to monitor the reactions. The PCR conditions consisted of one initial denaturing cycle at 95 °C for 5 min, followed by 35 cycles of 94 °C for 30s, 47 °C for 30 s, 72 °C for 45 s. This was followed by a final extension cycle at 72 °C for 7min.

These set of primers amplify a fragment of about 300–360bp of *Leishmania* genome (Schonian et al. 2003, Hajjaran et al. 2013). The PCR products were visualized after staining with ethidium bromide by 1.2% agarose gel electrophoresis in UV transluminator.

Molecular identification of *Leishmania* species was achieved by RFLP analysis. Ten microliters of the PCR product were added to 2μl of the enzyme buffer and 1μl of the HaeIII (BsuRI) Enzyme (Fermentas, Life Sciences, Germany), this mixture was incubated at 37 °C for 10 minutes, as recommended by the manufacturer. The cut site of enzyme was GG↓CC, producing different patterns based on *Leishmania* species. Digestion products were separated by using 3% agarose gels and visualized after staining by ethidium bromide.

## Results

**Fig. 2.**
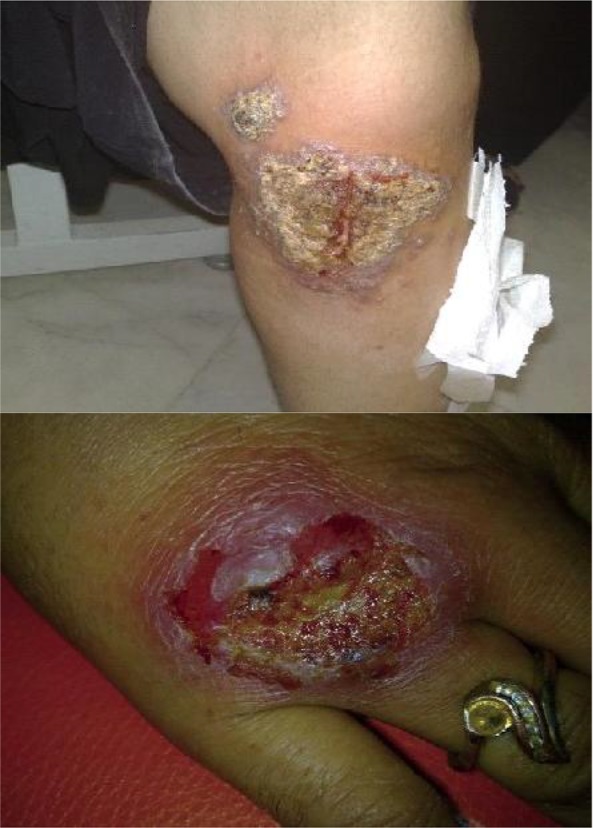
Representative pictures of skin lesions caused by *Leishmania major* in cutaneous leishmaniasis patients, Varamin, Iran, 2011–2012.

Totally, 44CL parasitologically positive patients were selected for molecular examination. The positive microscopic slides were scored for Leishman Body. The grading of *Leishmania* amastigotes numbers were as follow, 8% (1^+^), 42% (2^+^), 42% (3^+^) and finally 8% (4^+^). As Table 1 indicates the patients affected by CL were mostly from Bagher-Abad (27.3%) and Kheir-Abad (22.7%), nearly located in north-west of Varamin district near to Pakdasht. Among the patients, 63.6% were male. Patients ranged in age from 1 to 75yr old, the parasite infected all age groups except children under 1yr old and only 3 were above 65yr. The most highly infected age group was 25–39yr old (29.5%). Most of the patients (71%) had multiple lesions over exposed parts of body, even with 20 lesions in one case. Hands, legs and facial areas were the most affected parts of the body with 34.1%, 29.5% and 15.9%, respectively. Lesions were mostly of wet ulcerative type. The size of the most lesions was more than 1cm (Fig. 2). The average duration of lesions was 5 months; most of patients (99%) had no history of treatment.

### Molecular outcomes

**Fig. 3.**
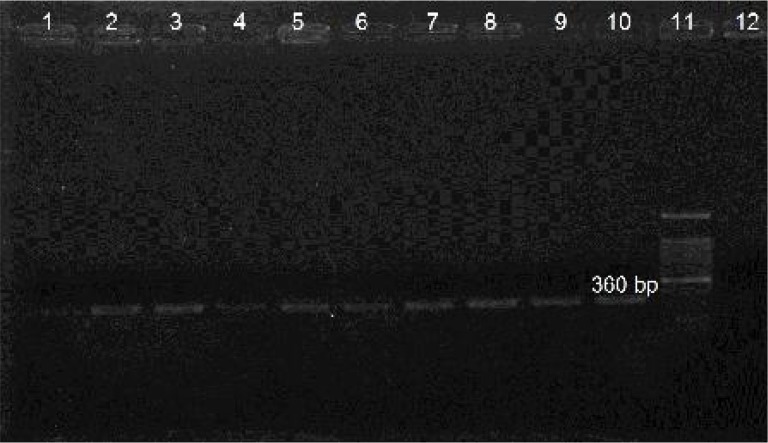
Electrophoresis of *Leishmania* DNA amplified with primers LITSR and L5.8S from exudate materials of lesions of suspected patients to cutaneous leishmaniasis, Varamin District, Iran, 2011–2012, Line 1–9: samples of patients with CL, line 10: *L. major* (Accession Number: JN860745), line 11: 100bp ladder marker (Fermentas) and line 12 negative control.

**Fig. 4.**
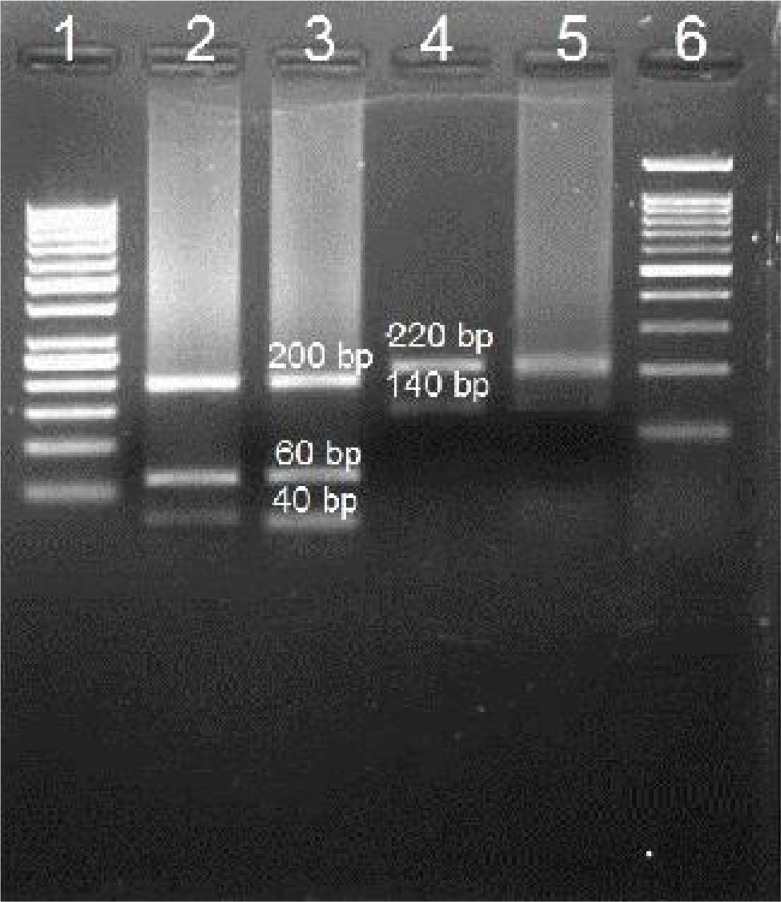
Digestion pattern of ITS1 amplicons of *Leishmania* spp of cutaneous leishmaniasis patients of Varamin District, by using HaeIII enzyme Line 1: 50 bp ladder marker, Line 2: sample of patients (*L. tropica*), Line 3: *L. tropica* (Accession. Noumber: EF653267), Line 4: *L. major* (Accession. Noumber: JN860745), Line 5 samples of patients (*L. major*), Line 6: 100 bp ladder marker

Using the LITSR/L5.8S primers set, a single product (300–360bp) has been amplified in all 44 samples (100%) (Fig. 3), the same as what observed for standard strains of parasite. After using the restriction enzyme, totally two bands of 220bp and 140bp were observed in 37 (84.1%) samples which was similar to what expected for *L. major* as it could be seen also in standard sample. They were mostly from Bagher-Abad (27.3%), Kheir-Abad (22.7%) and Varamin (18.2%) and the rest was scattered among 8 remained areas (Table 1). Besides, in 7 cases (15.9%), three bands of 200, 60 and 40bp were observed, comparing to standard strains, they are representative of *L*. *tropica* (Fig. 4). These cases were distributed in different areas as follows, 2 cases in Bagher-Abad, 2 in Kheir-Abad, 2 in Varamin and just one case in Reyhan-Abad. Table 1 showed more detailed in formation for *Leishmania* spp which were identified by PCR-RFLP.

## Discussion

Recently, the reported cases especially that of CL due to *L. major* have increased (Yaghoobi-Ershadi et al. 2010, Yaghoobi-Ershadi 2012, Hajjaran et al. 2014), and they were reported in areas known to be non-endemic or regions with decreasing trend over the passing years. Although Varamin is one of the old known foci of CL (Ardehali et al. 1994), but comprehensive data on CL was not available. A preliminary and purely parasitological study was done by the same authors who reported confirmed parasitological cases during 2012–2013 (Behravan et al. 2015). The evidence obtained by mentioned study indicates that Varamin district potentially encountered with ZCL. Therefore, ITS1-PCR-RFLP was conducted to identify the parasite species in patients. Entomological survey for vector incrimination was done in studied area by the same authors (Data under assay).

Totally among 44 CL patients, frequency of male was higher than of female. Some other endemic regions of the country have the same pattern, such as Damghan and Kashan (Rafati et al. 2007, Talari et al. 2006, Khosravi et al. 2013). It could be due to more contact of men with vectors based on the type and time of their work compared with women. Conversely, in Kerman Province, CL was distributed more significantly in females (Sharifi et al. 2008). Some studies have shown an equal distribution of ZCL infection among two sexes (Fakoorziba et al. 2011). Although most age groups were at risk of the disease, but based on obtained results, the prevalence of CL was higher in age group 25–39yr old. In known ZCL endemic regions of Iran, the highest risk group is often children less than 15yr old, in Kerman (< 10yr old), Fars Province (≤ 10 yr old), Damghan (10–19 yr) and part of Isfahan (under 1 yr of age) (Rafati et al. 2007, Sharifi et al. 2008, Nateghi Rostami et al. 2013). The effect of age might be actually influenced by disease endemicity and immune reactions of the host.

In this study, most of the lesions appeared on the extremities, this pattern is common in ZCL foci such as Damghan (Rafati et al. 2007) and Orzoieh district (Sharifi et al. 2008). Over half of the patients in current study had more than one lesion similar to what observed by other researchers in ZCL foci in Iran (Rafati et al. 2007, Sharifi et al. 2008). In CL due to *L. major*, multiple lesions are common (Yaghoobi-Ershadi et al. 2003). It could be because sand flies usually have a discontinuous blood-sucking habit, may bite several times and cause the development of several lesions on the skin (Yaghoobi-Ershadi et al. 2003, Yaghoobi-Ershadi et al. 2010, Nateghi Rostami et al. 2013).

All 44 available exudate materials which were confirmed positive by microscopic method were positive also by molecular tools (100%), which could be dedicated that the applied genetic marker and molecular method is quit useful, although it was not the aim of this study. *Leishmania major* as the main causative agent of ZCL was identified in most lesions (84.1%), which was not far from our expectation. About 80% of cases reported in the country are of ZCL form (Yaghoobi-Ershadi 2012). Same observations were reported from neighboring provinces of Varamin such as Semnan and Qom, which *L. major* was identified as the dominant causative agents of leishmaniasis in both areas (Rafati et al. 2007, Nateghi Rostami et al. 2013).

One the most important factors in leishmaniasis spreading are migration and travelling (Yaghoobi-Ershadi 2012). Varamin is the passage-way to the several large cities, especially religious city which caused a lot of people travel around. As a matter of fact, Varamin located very close to the capital of Iran, is one of the most important agricultural and animal husbandry axis, which caused it to be a good site for job seekers and consequently a lot of migration happen which increase the potential risk of disease.

## Conclusion

The achieved results, specifically dominancy of *L. major*, indicate that Varamin district could be regarded as one of the ZCL region in Iran, but to identify all epidemiological aspects of disease, a comprehensive epidemiological study is highly recommended.
